# Muscle oxygenation, endocrine and metabolic regulation during low-intensity endurance exercise with blood flow restriction

**DOI:** 10.20463/pan.2020.0012

**Published:** 2020-06-30

**Authors:** Hyejung Hwang, Sahiro Mizuno, Nobukazu Kasai, Chihiro Kojima, Daichi Sumi, Nanako Hayashi, Kazushige Goto

**Affiliations:** 1 Graduate school of Sport and Health Science, Ritsumeikan University, Shiga Japan; 2 Department of Physical Education, Hanyang University, Seoul Korea; 3 Physical Activity and Performance Institute (PAPI), Konkuk University, Seoul Korea; 4 Research Center of Health, Physical Fitness and Sports, Nagoya University, Nagoya Japan; 5 Department of Sports Science, Japan Institute of Sports Sciences, Tokyo Japan; 6 Research Center for Urban Health and Sports, Osaka City University, Osaka Japan

**Keywords:** low-intensity exercise, blood flow restriction, muscle oxygenation, endocrine response, energy metabolism

## Abstract

**[Purpose]:**

The present study investigated the effect of endurance exercise with blood flow restriction (BFR) performed at either 25% maximal oxygen uptake ($\dot{\text{V}}{\text{O}}_{\text{2}}$ max) or 40% $\dot{\text{V}}{\text{O}}_{\text{2}}$ max) on muscle oxygenation, energy metabolism, and endocrine responses.

**[Methods]:**

Ten males were recruited in the present study. The subjects performed three trials: (1) endurance exercise at 40% $\dot{\text{V}}{\text{O}}_{\text{2}}$ max without BFR (NBFR40), (2) endurance exercise at 25% $\dot{\text{V}}{\text{O}}_{\text{2}}$ max with BFR (BFR25), and (3) endurance exercise at 40% $\dot{\text{V}}{\text{O}}_{\text{2}}$ max with BFR (BFR40). The exercises were performed for 15 min during which the pedaling frequency was set at 70 rpm. In BFR25 and BFR40, 2 min of pressure phase (equivalent to 160 mmHg) followed by 1 min of release phase were repeated five times (5 × 3 min) throughout 15 minutes of exercise. During exercise, muscle oxygenation and concentration of respiratory gases were measured. The blood samples were collected before exercise, immediately after 15 min of exercise, and at 15, 30, and 60 minutes after completion of exercise.

**[Results]:**

Deoxygenated hemoglobin (deoxy-Hb) level during exercise was significantly higher with BFR25 and BFR40 than that with NBFR40. BFR40 showed significantly higher total-hemoglobin (total-Hb) than NBFR40 during 2 min of pressure phase. Moreover, exercise-induced lactate elevation and pH reduction were significantly augmented in BFR40, with concomitant increase in serum cortisol concentration after exercise. Carbohydrate (CHO) oxidation was significantly higher with BFR40 than that with NBFR40 and BFR25, whereas fat oxidation was lower with BFR40.

**[Conclusion]:**

Deoxy-Hb and total Hb levels were significantly increased during 15 min of pedaling exercise in BFR25 and BFR40, indicating augmented local hypoxia and blood volume (blood perfusion) in the muscle. Moreover, low-and moderate-intensity exercise with BFR facilitated CHO oxidation.

## INTRODUCTION

In traditional training procedures aimed to increase muscular strength and muscle hypertrophy, exercise intensity above at least 70% of one repetition maximum (1RM) is commonly recommended^1^ However, high-intensity exercise entails the risk of injury due to excessive stress on muscle joints as well as connective tissues in untrained or older people. In contrast, low intensity exercise (e.g., 20% of 1RM) with blood flow restriction (BFR) has beneficial effects even with short periods of training^2-4^. In particular, resistance exercise with BFR is effective in improving muscle strength and muscle hypertrophy^5-8^.

Exercise with BFR augments local hypoxia in muscle. The lowered muscle oxygenation during exercise is expected to elicit erythropoiesis with subsequent increases in oxygen transport capacity^9^, capillary density, mitochondrial biosynthesis, and myoglobin level in the tissues^10-11^. These cascades are stimulated by increased expression of hypoxia-inducible factor-1 (HIF-1) and vascular endothelial growth factor (VEGF), which are two major factors involved in angiogenesis^12^.

Several studies have shown that low-intensity endurance exercise (30-40% $\dot{\text{V}}{\text{O}}_{\text{2}}$ max) with BFR increases oxygen uptake, heart rate, and metabolite levels during and after exercise compared with normal exercise without BFR^13-15^. However, the influence of endurance exercise with BFR and the difference in effects with respect to low and extremely low intensity exercise on muscle oxygenation and metabolic regulation is currently unknown.

Therefore, the purpose of the present study was to investigate the effects of endurance exercise with BFR performed at 25% $\dot{\text{V}}{\text{O}}_{\text{2}}$ max or 40% $\dot{\text{V}}{\text{O}}_{\text{2}}$ max on muscle oxygenation, energy metabolism, and endocrine responses.

## METHODS

### Subjects

Ten males (mean± standard deviation [SD] age: 24.7 ± 2.1 years, height: 171.2 ± 5.7 cm, and body weight: 68.0 ± 7.8 kg) were recruited for the present study. They were healthy and had regular physical activity (few days/week, e.g., resistance exercise, endurance exercise). However, none of the subjects were involved in any training program at the start of the study. All subjects were explained the purpose of experiment, procedures, and the potential risks of the study. A written informed consent was subsequently obtained from each participant. The present study was approved by the Ethics Committee for Human Experiments at Ritsumeikan University.

### Experimental design

All subjects visited our laboratory four times during the experimental period. At the first visit, an incremental pedaling test was conducted to assess maximal oxygen uptake ($\dot{\text{V}}{\text{O}}_{\text{2}}$ max) using an ergometer (Aerobike 75XLIII; Konami Corporation, Tokyo, Japan). From second through fourth visits, three experimental trials were performed in a random order. The three trials consisted of endurance exercise at 40% $\dot{\text{V}}{\text{O}}_{\text{2}}$ max without BFR (NBFR40), endurance exercise at 25% $\dot{\text{V}}{\text{O}}_{\text{2}}$ max with BFR (BFR25), and endurance exercise at 40% $\dot{\text{V}}{\text{O}}_{\text{2}}$ max with BFR (BFR40). At least 7 days were prepared among trials.

For BFR25 and BFR40, specially designed tourniquets (E20 Rapid Cuff Inflator and Rapid Version Cuff, Hokanson, USA) were used to apply pressure during exercise, and the tourniquets were inflated at 160 mmHg pressure. Necessary information to accustom the subjects with the device was shared during the preliminary session. The tourniquet was placed at the proximal site of the middle thigh, both legs.

### Blood flow restriction and exercise protocols

**Figure 1.**
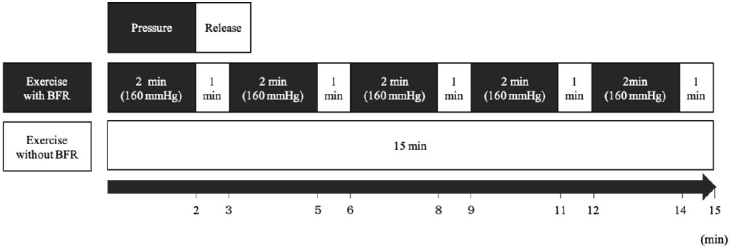
Exercise protocol with or without BFR

The tourniquet was designed to be 11 × 85 cm wide. It was used in conjunction with a rapid cuff inflator. The air inflator was controlled to maintain a stable level of required pressure (160 mmHg) during the pressure phase. Based on previous studies, we had set up 15 min pedaling exercise with a BFR protocol using an ergometer^4,32^. During the 15 min exercise in each trial, the pedaling frequency was set as 70 rpm. In BFR25 and BFR40, 2 min of pressure phase (equivalent to 160 mmHg) followed by 1 min of release phase were repeated five times (5 × 3 min) throughout the exercise. In NBFR40, the subjects wore a tourniquet, but no pressure was applied throughout the exercise (Fig. 1).

### Muscle oxygenation

During exercise, the muscle oxygenation level in the vastus lateralis muscle was evaluated noninvasively using near infrared spectroscopy (NIRS) (Hb14-2, Astem Co., Ltd. Kanagawa, Japan). The probe emitted two different wavelengths from the LED and photo diode, and detected the light transmitted through the body with the help of the light receiving element. The probe was placed on the right vastus lateralis (VL) muscle (at midpoint between the greater trochanter and lateral condyle of the femur), and the sampling rate was 10 Hz. The data were expressed as relative values to the baseline values obtained during rest. The oxygenated-hemoglobin (oxy-Hb), deoxygenated hemoglobin (deoxy-Hb) and total hemoglobin (total-Hb) levels were determined.

### Respiratory variables

Respiratory samples were collected using breath by breath method and analyzed using an automatic gas analyzer (AE300S, Minato Medical Science Co., Ltd., Tokyo, Japan) to determine $\dot{\text{V}}{\text{O}}_{\text{2}}$, carbon dioxide output ($\dot{\text{V}}\text{CO2}$), minute ventilation (VE), and the respiratory exchange ratio (RER). The carbohydrate and fat oxidation rates were also calculated from $\dot{\text{V}}{\text{O}}_{\text{2}}$ and $\dot{\text{V}}\text{CO2}$ using the following equations^16^. The collected data were averaged every 30 s.

### Exercise energy metabolism equations:

CHO oxidation(g/min) = 4.210 × $\dot{\text{V}}\text{CO2}$ −2.962 × $\dot{\text{V}}{\text{O}}_{\text{2}}$
FAT oxidation(g/min) = 1.695× $\dot{\text{V}}{\text{O}}_{\text{2}}$ −1.701× $\dot{\text{V}}\text{CO2}$
EE(kcal/15min) = 4.07×CHO oxidation + 9.75×FAT oxidation

### Blood sampling and analysis

Subjects arrived at the laboratory at approximately 8:00 AM following an overnight fast (at least 10 h after the previous meal). They rested about 20 min before the first blood collection. After rest, a 22-gauge polyethylene catheter was inserted into an antecubital vein and baseline blood sample was obtained. After exercise, subjects rested on the chair for an hour for blood collection. Blood samples were collected before exercise, immediately after 15 min of exercise, and at 15, 30, and 60 min after completion of exercise. Blood glucose and lactate concentrations were measured using a glucose analyzer (FreeStyle, Nipro Co., Osaka, Japan) and a lactate analyzer (Lactate Pro, Arkray Co., Kyoto, Japan) immediately after blood collection. The serum samples were obtained after 10 min of centrifugation at 4 ºC, and these samples were stored at -80 ºC until analysis. Serum growth hormone (GH), cortisol and myoglobin (Mb) concentrations were measured at a clinical laboratory (SRL, Inc., Tokyo, Japan). Heparin syringes (2.5 mL) were used to collect blood samples for determination of blood gas and electrolyte levels. From obtained blood samples, blood pH, HCO3−, base excess (BE), partial pressure of oxygen (pO2), partial pressure of carbon dioxide (pCO2), and sodium (Na+) and potassium (K+) concentrations were measured using an automatic blood-gas analyzer (OPTI CCA TS, Sysmex Co., Hyogo, Japan). Blood gas and electrolyte analyses were performed immediately after blood collection.

### Statistical analysis

All data are expressed as means ± SD. Time-dependent changes in variables were analyzed using two-way repeated measure analysis of variance (ANOVA) to confirm significant interaction (trial × time) and main effects for trial and time. When a significant interaction (time × trial) or main effect was detected, a post-hoc Tukey test was performed to identify differences. A P-value < 0.05 was considered to indicate statistical significance.

## RESULTS

### Muscle oxygenation

**Figure 2.**
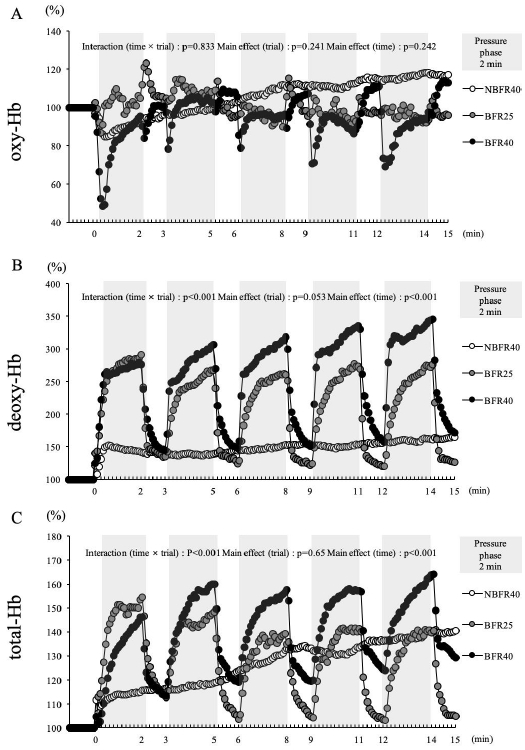
The percent changes of muscle oxygenation variables during exercise in the NBFR40, BFR40 and BFR25 every 5 s. (A) : The changes of oxygenated hemoglobin during exercise in the NBFR40, BFR25 and BFR40. (B) : The changes of deoxygenated hemoglobin during exercise in the NBFR40, BFR25 and BFR40. (C) : The changes of total hemoglobin during exercise in the NBFR40, BFR25 and BFR40 trials.

Figure 2 shows the relative changes in the variables of muscle oxygenation during 15 min of exercise. Oxy-Hb did not show a significant interaction (trial × time, p=0.83). Moreover, significant main effects of trial (p=0.24) and time (p=0.24) were not noted. The oxy-Hb level rapidly reduced during the pressure phase in BFR40, while NBFR40 revealed a slight increase in the over oxy-Hb level during the 15-min exercise session. Deoxy-Hb showed a significant interaction (trial × time, p<0.001), and the main effects of time (p<0.001) were noted. Although a marked increase in the deoxy-Hb levels were noted in during the pressure phase when exercise was performed with BFR (for the BFR25 and BFR40 trials), this decrease rapidly recovered during the subsequent release phase (1 min). In contrast, NBFR40 revealed slight elevation over 15 min of exercise. Total Hb showed a significant interaction (trial × time, p<0.001), and the main effects of time (p<0.001) were noted. The total-Hb levels increased during the pressure phase when exercise was performed with BFR (for the BFR25 and BFR40 trials), with a decrease in the levels during the subsequent release phase. In the NBFR40 trial, the total-Hb level gradually increased during the 15-min exercise session.

**Figure 3.**
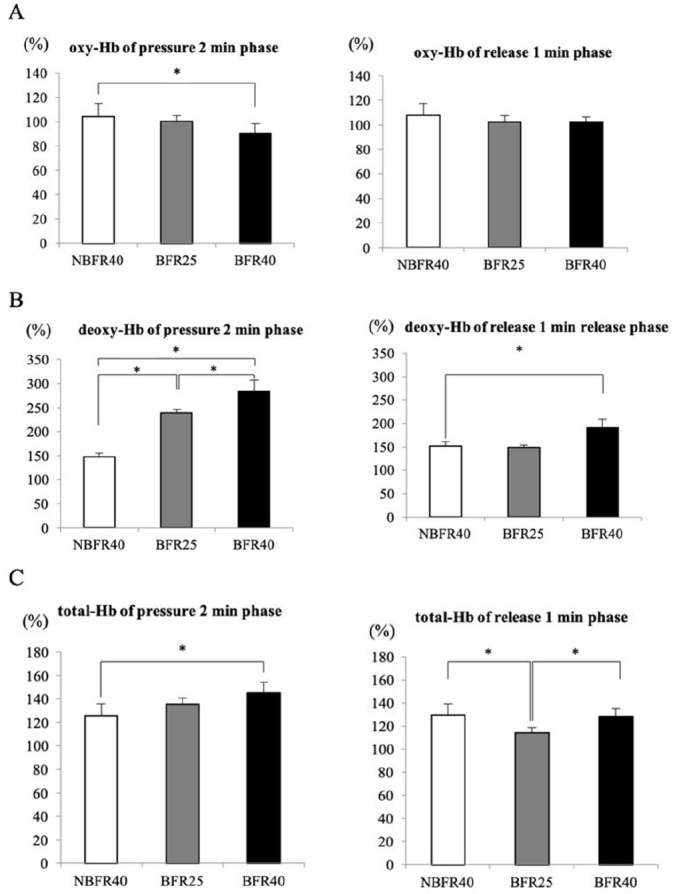
The percent changes of muscle oxygenation variables during 2 min pressure and 1 min release phase of 15min exercise in NBFR40, BFR25 and BFR 40. * *p*<0.05 between trials

Figure 3 presents the averaged muscle oxygenation variables during the pressure phase (2 min) and release phase (1 min) of the 15-min exercise session. The oxy-Hb level was significantly lower during the pressure phase in the BFR40 trial (p<0.05, Fig. 3A), while no significant difference was noted during the release phase. Particularly, the deoxy-Hb level was significantly increased during the pressure phase in the BFR25 and BFR40 trials. Furthermore, increased deoxy-Hb levels were noted during the release phase in the BFR40 trial (p<0.05, Fig. 3B). The total-Hb level significantly increased during the pressure phase in the BFR40 trial. During the release phase, the total-Hb level was significantly lower in the BFR25 trial than in the NBFR40 and BFR40 trials (p<0.05, Fig. 3C).

### Blood variables

Table 1 presents the changes in the blood variables before exercise and during the 60-min post-exercise period. The blood glucose, HCO3^−^, pO2, pCO2, Na^+^ and K^+^ concentrations did not differ significantly at any time point among the three trials. However, the blood lactate concentrations significantly increased after exercise only in the BFR40 trial (main effect for time, p<0.05), whereas a significant change was not observed over time in the NBFR40 and BFR25 trials. Immediately after exercise, the blood pH was significantly lower in the BFR40 trial than in the NFR40 and BFR25 trials. A significantly lower blood base excess was noted immediately after the 15-min exercise session in the BFR40 trial than in the BFR25 and NBFR40 trials.

**Table 1.** Changes in blood variables before exercise and during post-exercise period.

Variable	Trials	Pre	Post-exercise (min)			
			0	15	30	60
Glucose (mmol/L)	NBFR40	88.8 ± 3.9	81.8	85.0 ± 5.1	87.4 ± 4.7	83.1 ± 5.3
	BFR25	88.1 ± 4.8	86.8 ± 5.1	86.3 ± 3.9	85.6 ± 5.2	87.1 ± 5.5
	BFR40	89.2 ± 8.0	89.3 ± 10.0	94.3 ± 10.6	90.6 ± 9.7	88.9 ± 7.2
Lactate (mmol/L)	NBFR40	1.1 ± 0.3	1.5 ± 0.4	1.1 ± 0.2	1.1 ± 0.3	1.3 ± 0.2
	BFR25	1.3 ± 0.31	2.0 ± 0.5	1.5 ± 0.3	1.3 ± 0.3	1.4 ± 0.3
	BFR40	1.3 ± 0.3	5.2 ± 1.5 *#†	3.1 ± 1.0 *#†	2.3 ± 0.5 *#†	1.7 ± 0.4
pH	NBFR40	7.41 ± 0.01	7.41 ± 0.02	7.42 ± 0.01	7.42 ± 0.01	7.41 ± 0.02
	BFR25	7.42 ± 0.02	7.40 ± 0.02	7.42 ± 0.02	7.41 ± 0.01	7.41 ± 0.03
	BFR40	7.41 ± 0.02	7.36 ± 0.05*#†	7.39 ± 0.03	7.42 ± 0.04	7.41 ± 0.02
HCO_3_- (mmol/L)	NBFR40	27.3 ± 2.0	27.3 ± 1.8	27.5 ± 1.6	27.5 ± 1.3	26.4 ± 6.6
	BFR25	27.2 ± 1.3	26.6 ± 1.7	26.1 ± 1.9	27.3 ± 1.3	27.5 ± 1.3
	BFR40	26.8 ± 1.7	23.0 ± 2.2	23.8 ± 2.7	25.2 ± 2.5	27.1 ± 1.8
Base Excess (mmol/L)	NBFR40	2.3 ± 1.8	2.1 ± 1.6	2.6 ± 1.3	2.7 ± 0.9	2.9 ± 1.3
	BFR25	2.3 ± 1.4	1.4 ± 1.7	1.4 ± 1.7	2.3 ± 1.1	2.4 ± 1.0
	BFR40	1.9 ± 1.4	-2.5 ± 2.3*# †	-1.0 ± 2.4*#	0.8 ± 1.6	2.1 ± 1.7
PO_2_ (kPa)	NBFR40	8.45 ± 1.83	9.40 ± 1.66	8.87 ± 2.71	9.20 ± 1.85	6.53 ± 2.60
	BFR25	8.21 ± 2.31	8.84 ± 2.24	9.37 ± 2.65	5.77 ± 3.15	6.34 ± 1.55
	BFR40	9.14 ± 2.87	7.06 ± 1.84	9.06 ± 1.60	8.47 ± 2.14	7.88 ± 2.64
PO_2_ (kPa)	NBFR40	5.80 ± 0.32	5.93 ± 0.33	5.76 ± 0.36	5.75 ± 0.37	6.11 ± 0.43
	BFR25	5.73 ± 0.20	5.85 ± 0.28	5.52 ± 0.38	5.80 ± 0.26	5.91 ± 0.48
	BFR40	5.70 ± 0.45	5.63 ± 0.73	4.85 ± 1.81	5.37 ± 0.83	5.78 ± 0.37
Na+ (mmol/L)	NBFR40	138.7 ± 1.1	140.0 ± 1.4	138.8 ± 0.5	138.3 ± 1.1	138.7 ± 1.0
	BFR25	138.8 ± 1.6	139.1 ± 1.3	138.2 ± 1.1	138.7 ± 2.0	138.3 ± 1.1
	BFR40	137.9 ± 1.8	140.0 ± 1.5	138.9 ± 2.5	138.6 ± 2.0	138.6 ± 1.1
K+ (mmol/L)	NBFR40	3.65 ± 0.26	4.17 ± 0.19	3.83 ± 0.20	3.76 ± 0.27	3.50 ± 1.06
	BFR25	3.52 ± 0.15	3.96 ± 0.16	3.71 ± 0.15	3.69 ± 0.15	3.70 ± 0.11
	BFR40	3.49 ± 0.11	4.26 ± 0.38	3.72 ± 0.30	3.63 ± 0.12	3.76 ± 0.15

Values are presented as means ± SD. * Significant different from Pre ; p<.000, # Significant different from NBFR40 : p<0.05, † Significant different from BFR25 : p<0.05

### Serum GH, cortisol and myoglobin

**Figure 4.**
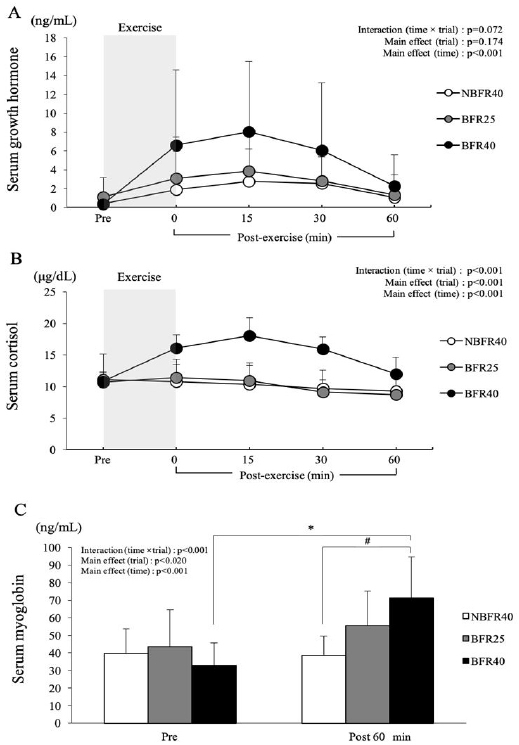
Exercise-induced changes in serum growth hormone (A), cortisol (B) and myoglobin concentrations (C) in NBFR40, BFR25 and BFR40. * Significant different from BFR40 at Pre ; p<.000, # Significant different from NBFR40 at Post 60 ; p<0.002, Values are presented as means ± SD.

Figure 4 presents the changes in the serum GH, cortisol and myoglobin concentrations. After exercise, the serum GH concentrations tended to be higher in the BFR40 trial than in the BFR25 and NBFR40 trials. However, there was no significant interaction (trial × time, p=0.07) or main effect for trial (p=0.17, Fig. 4A). The serum cortisol concentration showed a significant interaction (trial × time, p<0.001), and the main effects of trial (p<0.001) and time (p<0.001) were noted. Moreover, serum cortisol concentrations were significantly higher in the BFR40 trial than in the BFR25 and NBFR40 trial immediately after exercise and at 15 and 30 min after exercise (p<0.05, Fig. 4B). The serum myoglobin concentration showed a significant interaction (trial × time, p<0.001), and the main effect of time (p<0.001) was noted. In the BFR40 trial, the serum myoglobin concentration 60 min after the exercise session was significantly higher compared to that in the NBFR40 trial (p<0.05). However, no significant difference was observed between the NBFR40 and BFR25 trials (Fig. 4C).

### Energy metabolism during exercise

**Figure 5.**
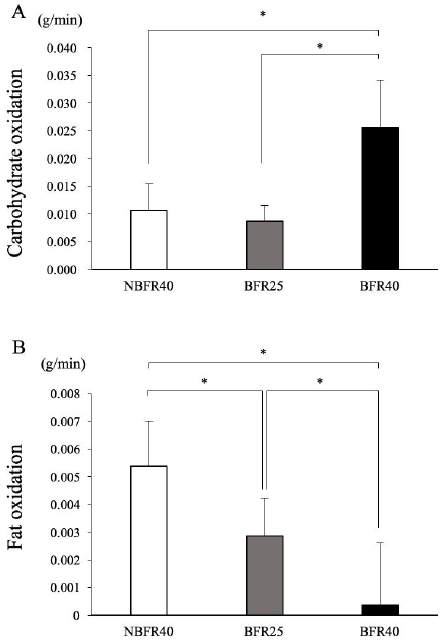
Carbohydrate (A) and fat oxidation (B) rate during 15 min of exercise. Values are presented as means ± SD. **p<0.05 between trials.

The averaged CHO and fat oxidation during the 15-min exercise session are presented in Fig. 5. CHO oxidation was significantly higher in the BFR40 trial than in the BFR25 and NBFR40 trials. Moreover, no significant difference was observed between the BFR25 and NBFR40 trials, although exercise intensity was different (25% $\dot{\text{V}}{\text{O}}_{\text{2}}$ max for BFR25 and 40% $\dot{\text{V}}{\text{O}}_{\text{2}}$ max for NBFR40, Fig. 3A). Fat oxidation was significantly lower in the BFR25 and BFR40 trials than in the NBFR40 trial. Furthermore, the lowest fat oxidation value among all the trials was noted in the BFR40 trial, and the value was significantly lower than those noted in the NBFR40 and BFR25 trials. The total energy expenditure during the 15-min exercise session was significantly lower in the BFR25 trial than in the NBFR40 and BFR40 trials (NBFR40: 99.7 ± 21 kcal; BFR25: 64.9 ± 16 kcal; BFR40: 112.1 ± 23.4 kcal, p<0.05). However, significant differences were not noted between the NBFR40 and BFR40 trials with respect to the total energy expenditure during the 15-min exercise session.

## DISCUSSION

The primary findings of the present study were that the deoxy-Hb level was significantly higher during the exercise session in the BFR25 and BFR40 trials than in the NBFR40 trial. A significantly higher total-Hb level was noted during the 2-min pressure phase in the BFR40 trial than in the NBFR trial. Moreover, exercise-induced increase in the lactate level and decrease in the pH were significantly higher in the BFR40 trial, with a concomitant increase in the serum cortisol concentration after exercise. Notably, the substrate oxidation pattern was altered with BFR during low-intensity endurance exercise. CHO oxidation was significantly higher in the BFR40 trial than in the NBFR40 and BFR25 trials, while fat oxidation was lower in the BFR40 trial. These findings suggest that BFR during low-intensity endurance exercise promotes muscle deoxygenation and CHO metabolism compared to that when the same exercise is performed without BFR.

During endurance exercise, the muscle blood flow is increased in response to the metabolic demands of the muscle^17,18^. NIRS is commonly used for evaluating the oxygenation levels and hemodynamics in a working muscle. As an individual starts the exercise, oxygen consumption and delivery to the skeletal muscle rapidly increases, up to 50-fold or more^19^. In the present study, significantly lower oxy-Hb levels were noted during the 2-min pressure phase in the BFR40 trial compared to those in the NBFR40 trial. Moreover, the deoxy-Hb and total-Hb levels were higher during the 2-min pressure phase and 1-min release phase in the BFR40 trial than in the NBFR40 trial. Several studies have reported that the oxy-Hb dissociation curve promoted the rate of deoxy-Hb at or close to the lactate and ventilatory thresholds [33, 34]. Moreover, the deoxy-Hb level during the pressure phase was significantly higher in the BFR25 trial than in the NBFR40 trial, despite lower exercise intensity in the BFR25 trial. The total-Hb level measured using NIRS reflects the blood volume in the muscle. As shown in Figure 3, the total-Hb level was significantly higher in the BFR40 trial than in the NBFR40 trial, suggesting that the cuff pressure during low-intensity endurance exercise augmented the muscle blood volume (blood perfusion). Endurance exercise with BFR augmented hyperemic blood flow in the local region, leading to increased shearing stress on the vascular endothelial cells^20^. Therefore, the augmented blood volume in the BFR40 trial may be because of the increased nitric oxide production induced by augmented shear stress^21-23^.

Notably, no difference was noted in the blood lactate levels or pH between the BFR25 and NBFR40 trials, despite the difference in the exercise intensity. In a previous study^24^, unilateral plantar flexion (30 repetitions/min) using 20% 1RM with BFR promoted a decrease in the in intramuscular phosphocreatine (PCr) and intramuscular pH, as measured by 31P-magnetic resonance spectroscopy (MRS), than exercise using 20% 1RM without BFR. Exercise with BFR induces metabolite accumulation and may affect endocrine response. In the present study, the serum GH level increased till 60 minutes after exercise in the BFR40 trial; however, but there was no significant difference in the serum GH levels among the trials. This result may be attributed to the short exercise duration (only 15 min). However, the serum cortisol concentration was significantly elevated in the BFR40 trial till 60 min after exercise; the serum cortisol and myoglobin concentrations were significantly elevated in the BFR40 trial at 60 min after exercise. Significant differences in the serum GH levels were not observed between the NBFR40 and BFR25 trials. Significant differences were not noted in the BFR40 trial; however, the serum GH tended to increase significantly in the BFR40 trial. Although the exercise-induced increase in the GH levels is dependent on the exercise intensity, the present findings suggest that BFR training effectively increases the serum GH levels. This may be an important finding with respect to prescribing exercise for untrained individuals, including elderly people.

In the present study, HR during the exercise was significantly increased in the BFR40 trial than in the NBFR40 and BFR25 trials (NBFR40: 106 ± 11; BFR25: 102 ± 13; BFR40: 137 ± 18, p <0.05). BFR stimulated autonomic cardiovascular (CV) response through a chemical stimulus of accumulation of metabolites and a mechanical stimulus, such as muscle exercise pressor reflex (EPR)^25^. The rating of perceived exertion (RPE) during the 2-min pressure phase was significantly higher in the BFR25 and BFR40 trials than in the NBFR40 trial (NBFR40: 1.6 ± 0.1; BFR25: 4.0 ± 0.4; BFR40: 5.8 ± 1.0, p<0.05). Stimulation of EPR through BFR may increase exercise-induced fatigue. Therefore, BFR during low-intensity endurance exercise augmented the score of subjective fatigue, probably owing to augmented central command^26,27^. Several studies have reported that endurance exercise with BFR enhanced the recruitment of the fast twitch fibers (FT fibers) during muscle activity. Enhanced FT fiber recruitment activates anaerobic glycolysis and alters the substrate oxidation pattern^4,28-30^. A 30-min low-intensity endurance exercise session with BFR increased CHO metabolism^13^. The muscle glycogen content significantly decreased after low-intensity resistance exercise with BFR compared to that when the same exercise is performed without BFR^31^. Our results indicate that CHO oxidation did not differ between the BFR25 and NBFR40 trials, despite the difference in the exercise intensity. The exercise intensity was same between the BFR40 and NBFR40 trials; however CHO oxidation was higher in the BFR40 trial. Therefore, we found that low-intensity exercise with BFR altered energy substrate utilization during exercise.

The present study has several limitations. Firstly, we did not evaluate the long-term training effects (e.g., change in the muscle strength, endurance, and muscle volume). Secondly, the present study recruited only healthy young male subjects. Although we applied the same pressure of 160 mmHg , the pressure intensity may vary depending on the muscle mass in the legs of the subjects. To clarify the benefit of low-and moderate-intensity exercise with BFR, further investigations in elderly people or clinical populations are required.

During the 15-min low-intensity (either 25% or 40% of $\dot{\text{V}}{\text{O}}_{\text{2}}$ max) endurance exercise session, the levels of deoxy-Hb and total Hb were significantly increased, when BFR was repeatedly applied. Moreover, lower levels of oxy-HB were noted during endurance exercise with BFR at 40% $\dot{\text{V}}{\text{O}}_{\text{2}}$ max achieved compared to those when the same exercise was performed without BFR. These findings suggest that BFR during low-intensity endurance exercise augmented local hypoxia and blood volume in the muscle. Furthermore, endurance exercise with BFR at 40% $\dot{\text{V}}{\text{O}}_{\text{2}}$ max promoted exercise-induced acidification in the blood (i.e., lower pH and higher blood lactate levels) compared to that when the same exercise was performed without BFR. Finally, BFR during low-intensity endurance exercise augmented CHO oxidation and impaired fat oxidation. Although the present study was performed as an acute experiment, the findings may suggest the spotential benefits of BFR during low-intensity endurance exercise for health promotion.

